# Temporal modulation improves dynamic peripheral acuity

**DOI:** 10.1167/19.13.12

**Published:** 2019-11-20

**Authors:** Jonathan A. Patrick, Neil W. Roach, Paul V. McGraw

**Affiliations:** Jonathan.Patrick1@nottingham.ac.uk; Neil.Roach@nottingham.ac.uk; Paul.McGraw@nottingham.ac.uk; Nottingham Visual Neuroscience, The University of Nottingham, Nottingham, UK; Nottingham Visual Neuroscience, The University of Nottingham, Nottingham, UK; Nottingham Visual Neuroscience, The University of Nottingham, Nottingham, UK

**Keywords:** *temporal modulation*, *visual acuity*, *dynamic vision*, *peripheral vision*, *psychophysics*

## Abstract

Macular degeneration and related visual disorders greatly limit foveal function, resulting in reliance on the peripheral retina for tasks requiring fine spatial vision. Here we investigate stimulus manipulations intended to maximize peripheral acuity for dynamic targets. Acuity was measured using a single interval orientation discrimination task at 10° eccentricity. Two types of image motion were investigated along with two different forms of temporal manipulation. Smooth object motion was generated by translating targets along an isoeccentric path at a constant speed (0–20°/s). Ocular motion was simulated by jittering target location using previously recorded fixational eye movement data, amplified by a variable gain factor (0–8). In one stimulus manipulation, the sequence was temporally subsampled by displaying the target on an evenly spaced subset of video frames. In the other, the contrast polarity of the stimulus was reversed at a variable rate. We found that threshold under object motion was improved at all speeds by reversing contrast polarity, while temporal subsampling improved resolution at high speeds but impaired performance at low speeds. With simulated ocular motion, thresholds were consistently improved by contrast polarity reversal, but impaired by temporal subsampling. We find that contrast polarity reversal and temporal subsampling produce differential effects on peripheral acuity. Applying contrast polarity reversal may offer a relatively simple image manipulation that could enhance visual performance in individuals with central vision loss.

## Introduction

The ability to resolve spatial detail declines dramatically as the retinal eccentricity of a visual target is increased (e.g., Brown, 1972a, 1972b; Demirel, Anderson, Dakin, & Thibos, 2012; Johnson, Keltner, & Balestrery, 1978). This fall off in spatial acuity, coupled with increasing amounts of visual crowding (Coates, Chin, & Chung, 2013; Hussain, Webb, Astle, & McGraw, 2012; Toet & Levi, 1992), places performance limits on a number of important visual tasks, such as reading ability (Battista, Kalloniatis, & Metha, 2005; Chung, Mansfield, & Legge, 1998; Pelli et al., 2007) and facial recognition (Mäkelä, Näsänen, Rovamo, & Melmoth, 2001; Rovamo, Mäkelä, Näsänen, & Whitaker, 1997). These limitations have particular functional consequences for individuals with central visual loss. Age-related loss of foveal sensitivity is the leading cause of visual disability and blind registration in the UK, Europe, and North America. Around 5% of the UK population over 65 years old are affected by age-related macular degeneration, with this figure increasing rapidly in older age groups (Owen et al., 2012). The central visual loss associated with macular disease is often profound and permanent, resulting in patients developing alternative viewing strategies. Typically, a peripheral pseudo-fovea, or preferred retinal locus (PRL) is developed at a more eccentric retinal location and is used to support spatial vision (Crossland, Culham, Kabanarou, & Rubin, 2005; Crossland, Engel, & Legge, 2011; Fletcher & Schuchard, 1997; Schuchard, 2005). Patients adapt to using a consistent PRL when viewing isolated targets and can be trained to move their eyes to reposition the PRL when searching more complex displays (Janssen & Verghese, 2016). Despite this however, visual task performance in patients with macular disease remains poor (Fine & Peli, 1995; Fletcher, Schuchard, & Watson, 1999; Legge, Ross, Isenberg, & LaMay, 1992; Legge, Rubin, Pelli, & Schleske, 1985) and developing new methods of optimizing the use of the remaining visual field is crucial.

While there is a tendency for researchers and clinicians to focus on the ability to resolve static targets, instances in which images remain stationary on the retina are, in fact, very rare. Retinal motion is produced both when objects move in space relative to an observer and when the eyes themselves move. The impact of smooth object motion on visual sensitivity has been well characterized. While slow motion can improve visual acuity when a target is partially obscured (Ağaoğlu, Herzog, & Öğmen, 2012; Frisén, 2010; Khuu & Kalloniatis, 2015; Patrick, Roach, & McGraw, 2017), increases in speed are typically associated with a loss in acuity (e.g., Brown, 1972a, 1972b; Burr & Ross, 1982; Burr, Ross, & Morrone, 1986; Westheimer & McKee, 1975) and an accompanying shift in the contrast sensitivity function towards lower spatial frequencies (Kelly, 1979, 1985; Burr & Ross, 1982). Coupled with the loss of resolving capacity associated with peripheral viewing, these motion-related impairments make discrimination of fine spatial detail particularly challenging in patients with central vision loss. Macular disease patients often present with larger amplitude fixational eye movements—the small, involuntary shifts in gaze position during periods of attempted fixation (Kumar & Chung, 2014; Macedo, Crossland, & Rubin, 2011; Macedo, Nascimento, Gomes, & Puga, 2007). This fixational instability introduces stochastic motion of the image across the retina that varies in speed and direction and has been suggested to play a role in the range of visual loss these patients experience (Falkenberg, Rubin, & Bex, 2007; Macedo et al., 2007; Whittaker, Budd, & Cummings, 1988).

In the normal visual system, evidence suggests that fixational eye movements can have beneficial effects on visual performance. A primary function is to recenter to target of fixation to the most sensitive retinal area following slow oculomotor drifts (Nachmias, 1961). Another suggestion is that spatial jitter of an image on the retina acts to stimulate a wider range of photoreceptors and prevent stimulus fading due to neural adaptation (e.g., Ditchburn & Ginsborg, 1952; Martinez-Conde, Macknik, Troncoso, & Dyar, 2006; Stevens et al., 1976; Yarbus, 1967, but see Poletti & Rucci, 2010, 2016 for an alternative view). More recently, it has been argued that fixational eye movements shape visual perception by altering the spectral composition of visual input. Fixational drift redistributes the spatial power of static visual input into the temporal domain. This has the effect of attenuating low spatial frequencies, flattening the 1/f spatial spectrum of natural images and making it easier to resolve fine detail (Boi, Poletti, Victor, & Rucci, 2017; Kuang, Poletti, Victor, & Rucci, 2012; Rucci, Iovin, Poletti, & Santini, 2007; Rucci & Victor, 2015). Experimental support for this idea comes from studies comparing visual performance when image motion due to fixation is removed using retinal stabilization techniques. Image stabilization selectively impairs the visibility of high spatial frequency stimuli, particularly when embedded in low spatial frequency noise (Rucci et al., 2007; Boi et al., 2017). Stabilization has also been shown to impair discrimination of high contrast optotypes presented at spatial scales near the resolution limit (Ratnam, Domdei, Harmening, & Roorda, 2017). More recently, Ağaoğlu et al. (2018) have shown that fine orientation discrimination performance is optimal, not when retinal motion is zero or fully compensated (albeit with a temporal flickering), but when there is some residual motion. At coarser spatial scales, the relationship between visual performance and the degree of stabilization disappears, suggesting increased tolerance to image motion for lower frequency information (Badcock & Wong, 1990; Falkenberg et al., 2007; Watson et al., 2012). Therefore, residual motion may be particularly important in spatially demanding tasks that require high frequency mechanisms.

If temporal modulations introduced by fixational eye movements are beneficial for visual performance, might it be possible to gain additional improvement by manipulating the temporal characteristics of visual input directly? Viewing a stimulus intermittently (e.g., under stroboscopic lighting), introduces contrast energy across a range of temporal frequencies (e.g., Van Santen & Sperling, 1985). In principle, an observer could exploit this information when trying to detect or identify a target, provided it falls within the limits of their spatio-temporal contrast sensitivity function, or “window of visibility” (Watson, 2013; Watson, Ahumada, & Farrell, 1986).

In this paper we describe a series of experiments aimed at increasing spatial acuity for objects in the peripheral visual field. We first investigate the effects of two forms of temporal modulation on acuity for static and smoothly moving targets: temporal subsampling, in which the target is presented with intermittent blank intervals (Experiment 1); and contrast polarity reversal, whereby the luminance contrast of the target is periodically inverted (Experiment 2). We show that temporal subsampling improves visual acuity for targets moving at high speed but impairs visual acuity for static and slowly moving targets. On the other hand, contrast polarity reversal is beneficial to acuity across a wide range of speeds. Finally, we gauge the potential for practical application of this approach in patients with central vision loss, where increases in the amplitude of microsaccades and drift result in greater fixational instability (Kumar & Chung, 2014). Our findings indicate that the benefits of contrast polarity reversal are maintained under simulated conditions of high fixation instability (Experiment 3).

## Methods

### Observers

A group of 12 observers participated in the study, comprising one of the authors (JAP) and 11 individuals (seven men, five women) who were naïve to the specific aims and experimental hypotheses. Of this group, eight completed Experiment 1A (age *M* 22.8, *SD* 2.3 years), five completed Experiment 1B (age *M* 24.5, *SD* 1.9 years), eight completed Experiment 2A and 2B (age *M* 24.0, *SD* 2.3 years), and six completed Experiments 3A and 3B (age *M* 25.2, *SD* 2.8 years). All observers had normal or corrected-to-normal visual acuity (at least 0.0 logMAR, 20/20, 6/6 as measured by an ETDRS chart) and no history of eye disease. Experimental procedures were explained in full prior to data collection and all observers gave informed consent. The research was approved by the School of Psychology ethics committee at The University of Nottingham and adhered to the tenets of the sixth revision of the Declaration of Helsinki.

### Stimuli and procedure

Stimuli were generated using PsychoPy v1.83 (Peirce, 2007) on a Mac Mini (Apple, Inc., Cupertino, CA) and displayed on a gamma-corrected LaCie Electron 22blue IV 20 in. monitor (1280 × 1024 resolution; Seagate technology, Tigard, OR) with 75 Hz refresh rate (frame duration 13.3 ms). Participants sat upright and screen distance was fixed at 100 cm using a chinrest. At this distance, one pixel subtends approximately 0.0175°. The participants responded using a standard QWERTY keyboard.

Visual acuity was assessed using an orientation discrimination paradigm with Landolt Cs oriented along each of the four oblique axes, presented monocularly to the right eye. The ratio between the diameter of a Landolt C and the width of the critical detail (the gap) was fixed at 5:1. For all conditions, the target display period was 0.33 s (25 video frames). A blank screen was shown while waiting for a response, followed by an intertrial interval of one second. A central fixation cross (0.5° height) was displayed at all times on the mid-gray background (43.3 cd/m^2^).

**Figure 1:**
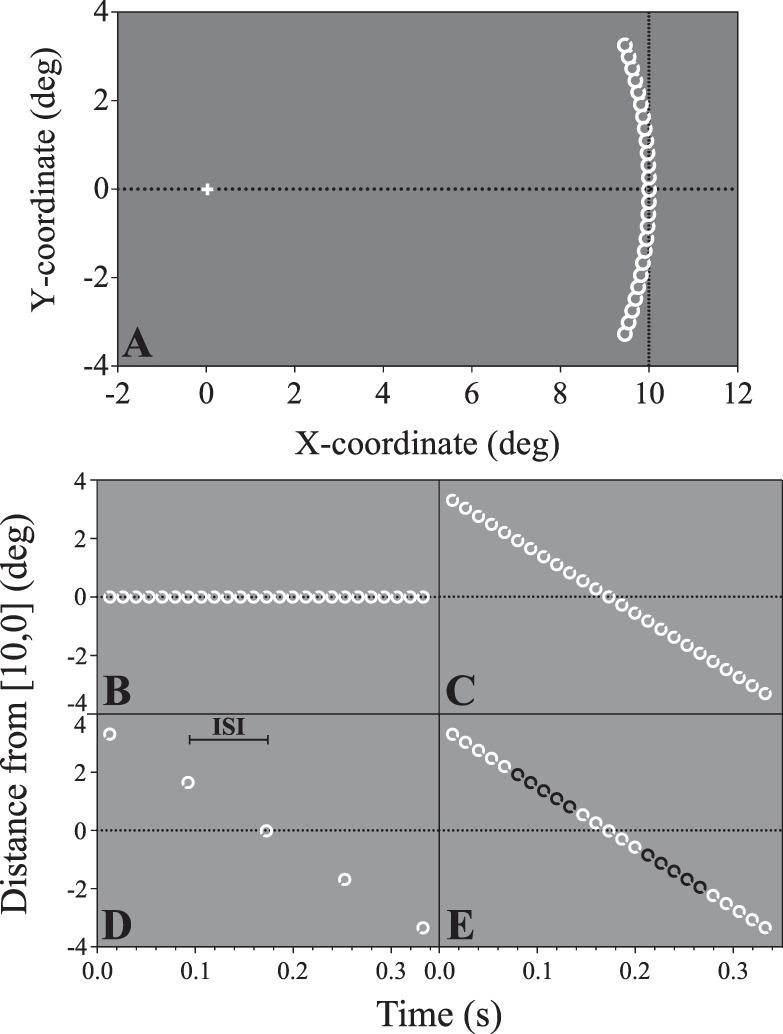
(A) Example of the stimulus trajectory in the smooth motion conditions (Experiments 1 and 2, to scale). The target follows an isoeccentric arc, 10° from fixation. This example shows the highest target speed (20°/s), presented in the right visual field. (B–E) Changes in stimulus position over time for the smooth object motion conditions, expressed in terms of the Euclidean distance of the target from the trajectory midpoint (10° from fixation on the horizontal meridian). Positive distance indicates the target is above the horizontal meridian, and negative distance below. (B) Space–time plot of a static target with no additional temporal modulation (0 ms ISI). (C) A target moving smoothly at 20°/s with no additional modulation. (D) Experiment 1: A target moving at 20°/s, temporally subsampled with 66.7 ms ISI. (E) Experiment 2: A target moving at 20°/s with the contrast polarity reversing at 66.7 ms intervals (after every fifth video frame). Note: The Landolt ring target is not shown to scale.

Targets in the smooth object motion conditions (Experiments 1 and 2) travelled along isoeccentric arcs centered on the horizontal meridian either 10° to the left or right of fixation. The side (left or right) and direction of travel (above the horizontal meridian to below, or vice versa) was randomized on each trial. Performance was assessed for static targets and five target speeds logarithmically spaced between 1.25°/s and 20°/s. Figure 1A and B show the target progression across one trial for static and moving targets without any additional temporal modulations, respectively.

**Figure 2:**
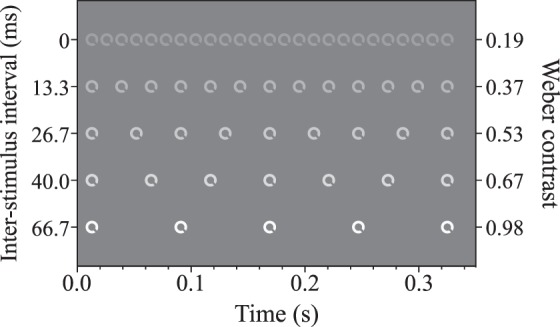
Graphical depiction of the contrast manipulation in Experiment 1B. The target in the higher subsampling conditions is presented at a lower contrast, such that the variance in the time-averaged Weber contrast between conditions was greatly reduced. The reported Weber contrast refers to the contrast of the target and background on a single frame, not the average for the condition.

In the first experiment, the target was presented at one of five different subsampling rates. In the smoothest (least subsampled) condition the target appeared on every video frame across the trial (0 ms ISI). As sample rate decreased, the number of frames on which the target appeared was reduced to every sixth video frame in the most subsampled condition (66.7 ms ISI, see Figure 1C). The target was presented at a luminance of 86.7 cd/m^2^ (Weber contrast 1.04). In Experiment 1A, the peak contrast was held constant across different subsampling conditions. However, reducing the number of frames on which the target was presented also lowered its time-averaged contrast. Therefore, in Experiment 1B we employed the complimentary control of covarying subsampling rate and peak contrast so as to equate time-averaged contrast. Target contrast in the least subsampled condition was reduced most, and so on. Target Weber contrast was set to 0.98/6 *p*, where *p* is the proportion of video frames on which the target was presented. For each condition, average Weber contrast was calculated by dividing the Weber contrast of the target by the number of frames on which it was presented. Changes to target luminance were designed to reduce the variance in the average contrast. Since target contrast was fixed between conditions in Experiment 1A, the range of average contrast was between 0.208 and 1.040 (*M* 0.491, *SD* 0.331). This range was reduced in Experiment 1B, to 0.188–0.196 (*M* 0.191, *SD* 0.003). The single-frame contrast of the target in each condition in Experiment 1B is shown in Figure 2.

In contrast polarity reversal conditions, the luminance of the target Landolt C was modulated in a square-wave fashion above and below the background luminance. In Experiment 2A, the full output range of the monitor was utilized, with luminance alternating between 0.1 cd/m^2^ and 86.7 cd/m^2^ (modulation depth = 1). Six alternation rates were examined: target luminance was reversed after periods ranging from 13.3 ms to 173.3 ms (i.e., alternating from every video frame to every 13 frames), alongside a control condition with no reversal. The contrast polarity on the initial frame was selected randomly on each trial. In Experiment 2B, the modulation depth was halved to 0.50 (high luminance 65.7 cd/m^2^, low luminance 20.9 cd/m^2^) and only the 66.7 ms reversal condition was examined.

Motion of targets in Experiment 3 was designed to mimic the effect of typical and exaggerated fixational eye movements. Offline, prior to the psychophysical procedure, fixational eye movements were recorded for each observer using an EyeLink 1000 eye tracker (SR Research, Ltd., Mississauga, Ontario, Canada), with 500 Hz binocular sampling rate and 30 arcmin average accuracy. The observers fixated on a 0.5° cross for three blocks of 10 s while eye position data were recorded. They were instructed to fixate on the cross and avoid blinking for the duration of each recording. Target locations for the experiments were extracted by downsampling recorded eye positions to match the 75 Hz refresh rate of the display monitor. Coordinates of eye positions were normalized to the median location, such that any overall spatial biases in the recording were removed.

**Figure 3:**
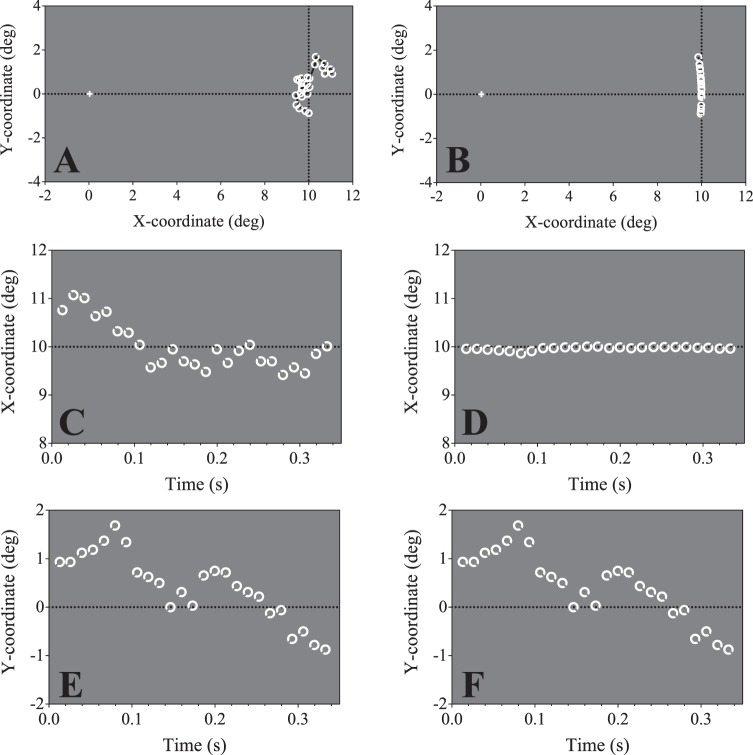
(A) Example target coordinates for the ocular motion conditions (Experiment 3A, to scale). The x- and y-coordinates were updated according to the eye-movement data of the participant. The retinal eccentricity of the target is therefore able to vary during each trial. The amplitude gain factor in this example is the maximum, 8, such that the size of the displacement of the stimulus from coordinates (10,0) reflects typical eye movements of the participant during fixation exaggerated by a factor of 8. (B) In Experiment 3B, the y-coordinate was updated on each frame according to the eye-movement data of the participant, and the x-coordinate was calculated to maintain a target eccentricity of 10°. The gain factor in this example is 8. (C–F) Changes in target positions from (A) and (B) across the trial duration. (C, E) show how the x- and y-coordinates of the target in (A) change across the trial. (D, F) show the x- and y-coordinates of the target in (B). Note the y-coordinate positions (E, F) are the same for both conditions, but the x-coordinates in Experiment 3B (D) serve to maintain a constant target eccentricity of 10°.

Deviations in eye position were applied to the target, multiplied by a variable gain factor of 0, 0.5, 1, 2, 4, or 8. Under this approach, a gain factor of zero resulted in a static target, a gain factor of 1 mimicked the jitter introduced by the natural eye movements of the observer, and gain factors above and below 1 exaggerated or attenuated the magnitude of image jitter, respectively. In Experiment 3A, a random time point from the eye-tracking data was chosen at the start of each trial, and the frame position of the stimulus was modified by the frame position of fixation at that time point. The *xy*-positions of the subsequent 24 time points were used for the remaining trial frames, such that the stimulus jitter followed the pattern of eye movements over the course of that duration during the fixation task, with 0 ms ISI. An example stimulus trajectory over the course of one trial for a gain factor of 8 is shown in Figure 3A.

A consequence of jittering the target stimulus in this way is that its distance from fixation varies over time. To ensure that changes in acuity associated with increased positional jitter was not due to changes in eccentricity, in Experiment 3B we varied the vertical position of the target using subjects' eye-tracking data, while adjusting the horizontal position so as to maintain a fixed eccentricity of 10°. An example of this manipulation is shown in Figure 3B. The space-time evolution of the targets in Experiment 3 is analogous to those in Figure 1, albeit with irregular between-frame shifts in displacement.

Three temporal modulation conditions were included in Experiments 3A and 3B. The unmodulated targets were presented at peak contrast (analogous to the unmodulated conditions in Experiment 1A and 2A), rather than the time-averaged contrast of Experiment 1B. As the reduced contrast condition is unlikely to provide the optimum characteristics in patient groups, we have not examined it further here. The contrast polarity reversal condition matched the temporal pattern of the 66.7 ms reversal condition in Experiment 2A, and the temporal subsampling condition matched that of the 66.7 ms ISI condition in Experiment 1A.

In each experiment, target size was manipulated using a 3-down–1-up staircase procedure. The Landolt C diameter was initially set to 1.5° and changed in steps of 0.4° Step size was then halved every second reversal. Observers were instructed to maintain fixation on the central cross throughout the trial. Following each stimulus presentation, observers reported the orientation of the Landolt C via a keyboard button press. They received immediate feedback for a correct or incorrect response. Staircases terminated after a minimum of eight reversals and 50 trials. Observers completed five staircases for each condition, giving a minimum of 250 trials. Testing was spread across several sessions, each lasting approximately one hour and occurring on different days.

### Analysis

For each observer and condition, the proportion of correct responses ($\newcommand{\bialpha}{\boldsymbol{\alpha}}\newcommand{\bibeta}{\boldsymbol{\beta}}\newcommand{\bigamma}{\boldsymbol{\gamma}}\newcommand{\bidelta}{\boldsymbol{\delta}}\newcommand{\bivarepsilon}{\boldsymbol{\varepsilon}}\newcommand{\bizeta}{\boldsymbol{\zeta}}\newcommand{\bieta}{\boldsymbol{\eta}}\newcommand{\bitheta}{\boldsymbol{\theta}}\newcommand{\biiota}{\boldsymbol{\iota}}\newcommand{\bikappa}{\boldsymbol{\kappa}}\newcommand{\bilambda}{\boldsymbol{\lambda}}\newcommand{\bimu}{\boldsymbol{\mu}}\newcommand{\binu}{\boldsymbol{\nu}}\newcommand{\bixi}{\boldsymbol{\xi}}\newcommand{\biomicron}{\boldsymbol{\micron}}\newcommand{\bipi}{\boldsymbol{\pi}}\newcommand{\birho}{\boldsymbol{\rho}}\newcommand{\bisigma}{\boldsymbol{\sigma}}\newcommand{\bitau}{\boldsymbol{\tau}}\newcommand{\biupsilon}{\boldsymbol{\upsilon}}\newcommand{\biphi}{\boldsymbol{\phi}}\newcommand{\bichi}{\boldsymbol{\chi}}\newcommand{\bipsi}{\boldsymbol{\psi}}\newcommand{\biomega}{\boldsymbol{\omega}}{p_{correct}}$), at each target gap size ($\newcommand{\bialpha}{\boldsymbol{\alpha}}\newcommand{\bibeta}{\boldsymbol{\beta}}\newcommand{\bigamma}{\boldsymbol{\gamma}}\newcommand{\bidelta}{\boldsymbol{\delta}}\newcommand{\bivarepsilon}{\boldsymbol{\varepsilon}}\newcommand{\bizeta}{\boldsymbol{\zeta}}\newcommand{\bieta}{\boldsymbol{\eta}}\newcommand{\bitheta}{\boldsymbol{\theta}}\newcommand{\biiota}{\boldsymbol{\iota}}\newcommand{\bikappa}{\boldsymbol{\kappa}}\newcommand{\bilambda}{\boldsymbol{\lambda}}\newcommand{\bimu}{\boldsymbol{\mu}}\newcommand{\binu}{\boldsymbol{\nu}}\newcommand{\bixi}{\boldsymbol{\xi}}\newcommand{\biomicron}{\boldsymbol{\micron}}\newcommand{\bipi}{\boldsymbol{\pi}}\newcommand{\birho}{\boldsymbol{\rho}}\newcommand{\bisigma}{\boldsymbol{\sigma}}\newcommand{\bitau}{\boldsymbol{\tau}}\newcommand{\biupsilon}{\boldsymbol{\upsilon}}\newcommand{\biphi}{\boldsymbol{\phi}}\newcommand{\bichi}{\boldsymbol{\chi}}\newcommand{\bipsi}{\boldsymbol{\psi}}\newcommand{\biomega}{\boldsymbol{\omega}}x$) was calculated and fitted with a logistic function of the form:

$$\newcommand{\bialpha}{\boldsymbol{\alpha}}\newcommand{\bibeta}{\boldsymbol{\beta}}\newcommand{\bigamma}{\boldsymbol{\gamma}}\newcommand{\bidelta}{\boldsymbol{\delta}}\newcommand{\bivarepsilon}{\boldsymbol{\varepsilon}}\newcommand{\bizeta}{\boldsymbol{\zeta}}\newcommand{\bieta}{\boldsymbol{\eta}}\newcommand{\bitheta}{\boldsymbol{\theta}}\newcommand{\biiota}{\boldsymbol{\iota}}\newcommand{\bikappa}{\boldsymbol{\kappa}}\newcommand{\bilambda}{\boldsymbol{\lambda}}\newcommand{\bimu}{\boldsymbol{\mu}}\newcommand{\binu}{\boldsymbol{\nu}}\newcommand{\bixi}{\boldsymbol{\xi}}\newcommand{\biomicron}{\boldsymbol{\micron}}\newcommand{\bipi}{\boldsymbol{\pi}}\newcommand{\birho}{\boldsymbol{\rho}}\newcommand{\bisigma}{\boldsymbol{\sigma}}\newcommand{\bitau}{\boldsymbol{\tau}}\newcommand{\biupsilon}{\boldsymbol{\upsilon}}\newcommand{\biphi}{\boldsymbol{\phi}}\newcommand{\bichi}{\boldsymbol{\chi}}\newcommand{\bipsi}{\boldsymbol{\psi}}\newcommand{\biomega}{\boldsymbol{\omega}}{p_{correct}} = 0.25 + {{0.75} \over {1 + {e^{{{\mu - x} \over \sigma }}}}}$$

The best-fitting free parameter *μ* was taken as an estimate of the resolution threshold, corresponding to the target gap size producing 62.5% correct performance (i.e., midway between chance and perfect performance); *σ* is a free parameter controlling the slope of the psychometric function.

Two-way, repeated measures analysis of variance (ANOVA) was performed on the group data for each experiment to assess the main effects of target speed and temporal modulation on thresholds. Where significant interaction effects indicated non-monotonic changes in relative effects of speed and modulation, further statistical analysis was performed on the improvement ratio for each modulation condition, to further quantify the effects of the additional temporal modulations. Each subject's resolution threshold for a given target speed and modulation condition was divided by the resolution threshold of the corresponding unmodulated condition. A two-tailed Student's *t* test was performed on each improvement ratio, testing the null hypothesis of no change in resolution threshold (improvement ratio = 1). Each planned test was performed at every target motion condition (i.e., six tests per family), so the alpha value for reporting statistical significance was reduced in accordance with Bonferroni correction to 0.0083. Statistically significant diversions from 1 in improvement ratios are identified with color-coded asterisks.

## Results

### Experiment 1

**Figure 4:**
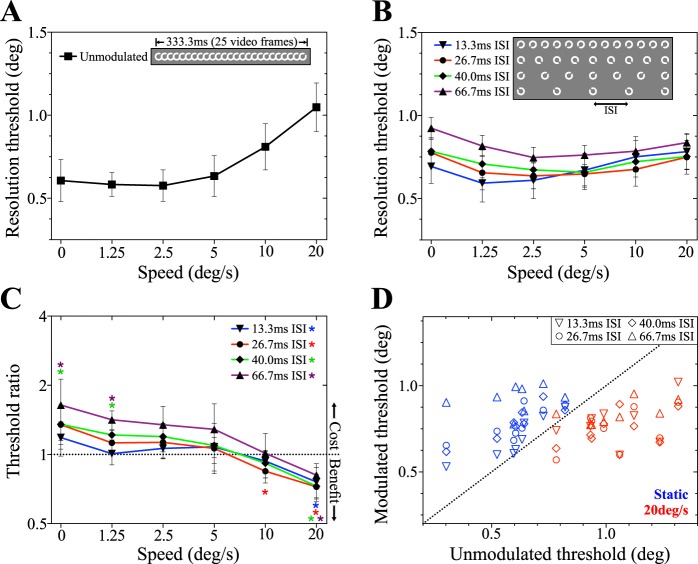
Results of Experiment 1A. Temporal subsampling affects resolution thresholds for smoothly moving peripheral targets across a range of speeds. (A) Resolution thresholds as a function of target speed for smoothly moving targets without additional modulation (target presentation on every video frame, as shown in inset). (B) Resolution thresholds as a function of target speed for temporally subsampled peripheral targets. Inset details number and spacing of frames on which the target was displayed in each condition. (C) The ratio of modulated to unmodulated resolution threshold for each ISI and speed. Values above the dotted line indicate subsampling reduced resolution compared to the unmodulated condition, and values below indicated enhanced resolution. Data points significantly above or below 1 are identified with asterisks, color-coded to the modulation condition. (D) Modulated and unmodulated resolution thresholds for individual subjects, for static targets (blue symbols) and the fastest moving targets (20°/s, red symbols). The dotted line indicates equal performance for unmodulated and modulated conditions. Points above the line indicate performance was better in the unmodulated condition, and below the line in the relevant modulation condition. Data points indicate the group or individual mean resolution thresholds and error bars are the between-subjects 95% confidence intervals (CI).

Mean resolution thresholds for smoothly moving targets are shown in Figure 4A. In the absence of any temporal modulation, increasing target speed results in a systematic rise in threshold, consistent with previous reports (Brown, 1972a, 1972b). As shown in Figure 4B, performance in subsampled conditions becomes progressively less speed-dependent with increasing ISI. Relative to performance in the non-modulated condition, this manifested as an improvement in performance at high speeds coupled with an impairment of performance at low speeds. This pattern can be clearly seen in Figure 4C, which plots the ratio of sampled to unsampled threshold for each stimulus speed along with 95% confidence intervals. To confirm the statistical significance of this result, we ran a two-way repeated measures analysis of variance (ANOVA) on the raw threshold scores. This revealed significant main effects of target speed, *F*(5, 35) = 26.04; *p* < 0.0001, and modulation condition, *F*(4, 28) = 7.29; *p* = 0.0004, as well as a significant interaction, *F*(20, 140) = 14.96; *p* < 0.0001. The results of *t* tests comparing modulated and unmodulated conditions at each speed and ISI are shown in Table A1, and significant differences are indicated by the asterisks marked on Figure 4C. Mean performance in the 40.0 ms and 66.7 ms ISI conditions was significantly worse than for unmodulated targets at 0°/s and 1.25°/s. In contrast, subsampling significantly improved performance in the 26.7 ms ISI condition at 10°/s, and all four modulation conditions at 20°/s. Figure 4D shows how individual subjects' modulated and unmodulated resolution thresholds compared for the static (0°/s) and fastest (20°/s) targets. For static targets, the data points are all above the dashed line, indicating higher thresholds for subsampled targets. For moving targets, all but one data point fall below the line, demonstrating consistent improvement in subsampled conditions within individual subjects.

**Figure 5:**
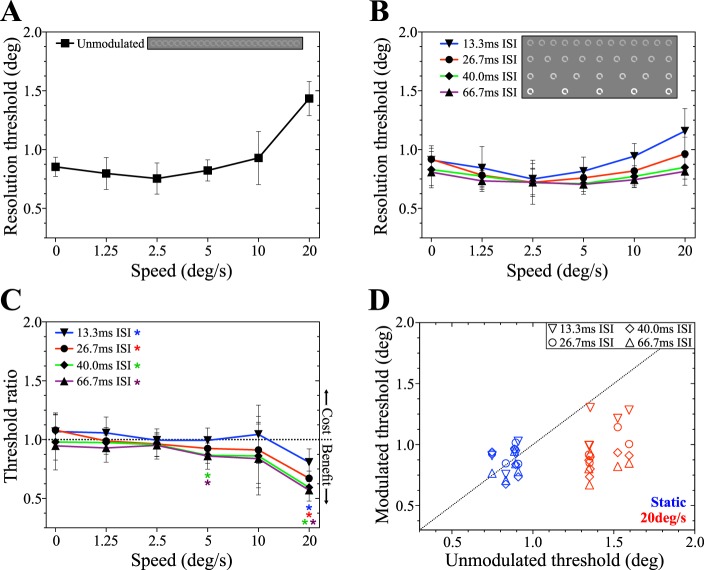
Results of Experiment 1B, examining the effect of contrast-controlled temporal subsampling on resolution thresholds across a range of target speeds. (A) Resolution thresholds as a function of target speed for the unmodulated condition (0 ms ISI). (B) Thresholds as a function of speed for each of the conditions with additional temporal modulations. Inset shows the number of video frames on which the target was presented in each condition. (C) Ratio of modulated to unmodulated resolution thresholds, for each of the four subsampling conditions. The ordinate shows the modulated threshold divided by the unmodulated threshold. The dashed line reflects no change. Points above indicate a detrimental effect of subsampling, points below show that subsampling improved resolution. (D) Individual subjects' resolution thresholds for temporally modulated targets as a function of their corresponding unmodulated resolution threshold, for static targets (blue symbols) and the fastest examined targets (20°/s, red symbols). Points above the dotted line indicate subsampling was detrimental to resolution. Error bars are between-subjects 95% CI.

Figure 5 shows the results of Experiment 1B, in which we controlled for the progressive reduction in time-averaged contrast experienced in subsampled conditions (see Methods and Figure 2 for details). The detrimental effect of increasing speed was replicated for unsampled targets presented at a lower contrast (Figure 5A) as was the reduction in the effect of speed once subsampling was introduced (Figure 5B). A two-way, repeated measures ANOVA revealed significant main effects of speed, *F*(5, 20) = 30.97; *p* < 0.0001, and modulation condition, *F*(4, 16) = 23.86; *p* < 0.0001, as well as a significant interaction, *F*(20, 80) = 10.69; *p* < 0.0001. However, the pattern of results driving the interaction effect was noticeably different to Experiment 1A. Specifically, while subsampling improved performance at high speeds, no systematic detriment to performance was observed at slower speeds (see Figure 5C, Figure 5D). Full results of *t* tests comparing mean thresholds in subsampled and unsampled conditions are shown in Table A2.

### Experiment 2

Although temporal subsampling improved acuity at high speeds in Experiment 1, it was either detrimental (Experiment 1A) or had little influence (Experiment 1B) at low speeds, limiting its potential practical utility as a means of improving peripheral function. In Experiment 2, we investigated whether contrast polarity reversal can provide more general benefits for peripheral visual acuity.

**Figure 6:**
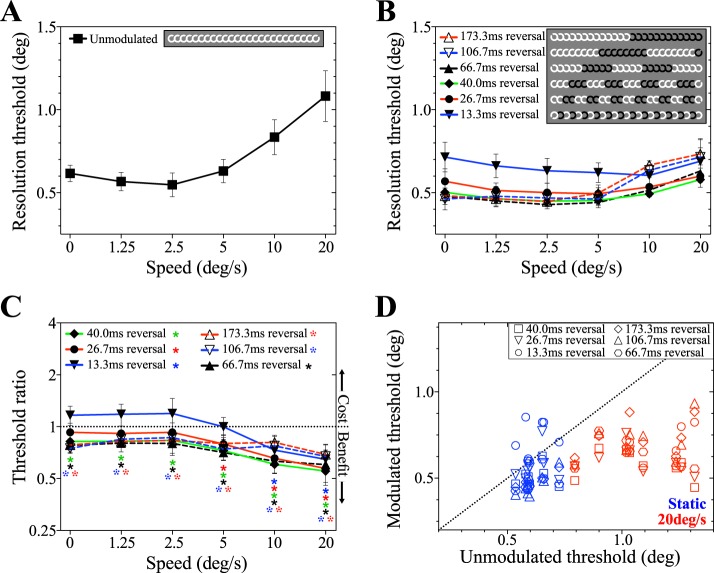
Results of Experiment 2A, showing the effect of contrast polarity reversal on resolution of peripheral targets. (A) Resolution thresholds for smoothly moving peripheral targets without additional temporal modulation (no reversal of contrast polarity). (B) Resolution thresholds as a function of target speed for targets with periodically reversing contrast polarity. Inset details the number of consecutive frames on which the target was presented before contrast polarity reversed. (C) Improvement ratio for polarity reversal conditions as a function of target speed. The ordinate shows threshold for modulated stimuli divided by the unmodulated threshold for that speed. Data above 1 (dotted line) indicate poorer performance in the modulated condition, data below 1 show better performance. Significant diversions from 1 are identified with asterisks. (D) Individual subjects' resolution thresholds for static targets (top) and targets moving at 20°/s (bottom). Modulated threshold (with contrast polarity reversal) is on the ordinate and unmodulated (without) on the abscissa. Data above the diagonal suggest resolution was better in the unmodulated condition, data below the diagonal suggest the opposite. Data are mean resolution thresholds, and error bars are between-subjects 95% CI.

Figures 6A and 6B show mean resolution thresholds plotted as a function of speed for unmodulated and contrast modulated targets respectively. Similar to our results with temporal subsampling, performance in contrast polarity reversal conditions showed little or no speed-related decline (Figure 6B); thresholds in the conditions with reversal periods between 13.3–66.7 ms do not rise as speed increases, whereas the conditions with 106.7 ms and 173.3 ms reversal periods show a moderate rise above 5°/s. Resolution thresholds in the fastest reversal condition (13.3 ms, or reversal after every video frame) are higher than the other conditions. The main effects of target speed and modulation condition were assessed with a two-way, repeated measures ANOVA. Both main effects of speed and modulation were significant, *F*(5, 35) = 84.76; *p* < 0.0001, and *F*(6, 42) = 26.39; *p* < 0.0001, respectively. A significant interaction effect was also found, *F*(30, 210) = 16.59; *p* < 0.0001, reflecting a nonuniform effect of polarity reversal rate across the range of speeds tested. As shown in Figure 6C, this is primarily driven by a lack of threshold enhancement at low speeds in the 13.3 ms polarity reversal condition. Results of *t* tests comparing mean resolution thresholds in modulated and unmodulated conditions are shown in Table A3. The relationship between individual subjects' modulated and unmodulated thresholds for static targets and targets moving at 20°/s is shown in Figure 6D.

**Figure 7:**
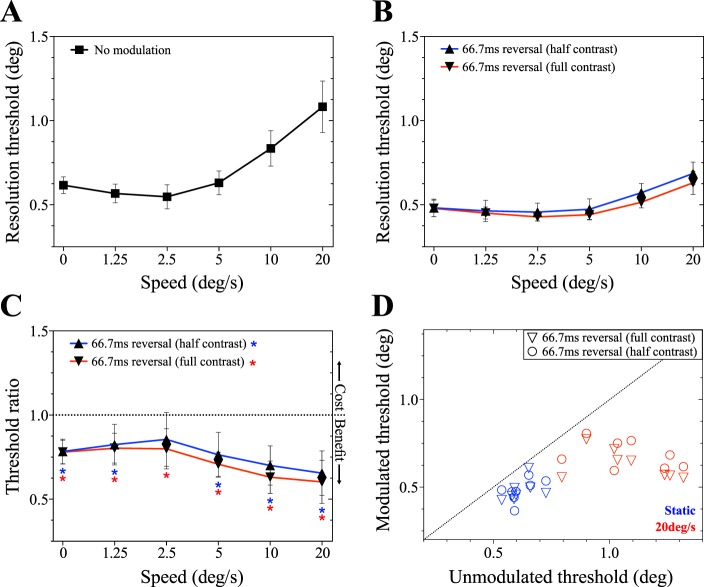
Results of Experiment 2B. (A) Resolution thresholds for targets moving smoothly along isoeccentric peripheral arcs as a function of target speed. (B) Resolution thresholds for targets with reversing contrast polarity at full- and half-maximum contrast range. (B) The ratio of modulated to unmodulated resolution thresholds for full- and half-maximum contrast polarity reversal conditions. Dotted line indicates no effect. (D) Individual subjects' resolution thresholds for modulated conditions as a function of the corresponding unmodulated resolution threshold for 0°/s and 20°/s. Dotted line indicates equal modulated and unmodulated thresholds.

Experiment 2A demonstrates that contrast polarity reversal enhances peripheral resolution for static and moving targets. This is, however, confounded by a reduced contrast range in the control (unmodulated) condition: the maximum contrast in the unmodulated trials is the white target against the mid-gray background, while in the modulated conditions, it is the white target against the black target. Accordingly, in Experiment 2B the contrast range of the reversal condition was halved. Resolution thresholds as a function of speed are compared for unmodulated targets, targets reversing periodically with the full contrast range, and targets reversing with the reduced contrast range in Figure 7.

The reduction in resolution thresholds when targets reverse contrast polarity (Experiment 2A) was also found in Experiment 2B (Figure 7A). Both full- and half-maximum contrast reversal produced resolution thresholds lower than those obtained with unmodulated targets. This is confirmed in Figure 7B, wherein all data points are below the dashed line, indicating that contrast polarity reversal enhances resolution. This enhancement is significant for all speeds in the full contrast condition, and in all except for 2.5°/s in the half contrast condition (Table A4). Threshold ratios for the full and half contrast conditions were also tested against each other but were not significantly different at any speed (Table A4).

### Experiment 3

Patients with central vision loss often demonstrate exaggerated fixational eye movements compared to healthy eyes (Kumar & Chung, 2014). We next sought to verify whether the benefits of contrast polarity reversal demonstrated in the previous experiment generalize to retinal motion conditions associated with poor fixation stability. To do this, we recorded subjects' fixational eye movements and then applied them to the target position with a variety of gain factors (see Methods for details). In Experiment 3A, target position was allowed to vary in both vertical and horizontal directions without restriction, whereas in Experiment 3B, target position was constrained to an iso-eccentric arc, 10° from fixation (Figure 3).

**Figure 8:**
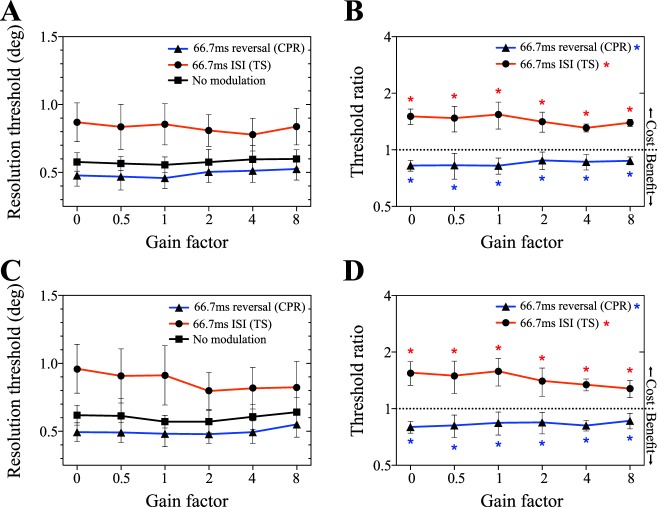
(A) and (B) show the results of Experiment 3A, examining resolution of peripheral targets moving along trajectories mimicking ocular motion. In this experiment, target eccentricity was unrestrained; the simulated retinal instability was applied to both the x- and y-coordinates (cf. Figure 3A and B). (A) Resolution thresholds as a function of gain factor for each of the three temporal modulation conditions. (B) Modulated to unmodulated threshold ratio for each of the applied temporal modulation conditions. Data points below 1 (dotted line) have lower resolution thresholds than in the unmodulated condition; data points above 1 imply a detrimental effect. (C) and (D) show the resolution thresholds and threshold ratios for Experiment 3B, respectively. Targets were constrained to an isoeccentric arc, 10° from fixation. The y-coordinate was drawn from observers' fixation data, and the x-coordinate was calculated to maintain the constant eccentricity (cf. Figure 3C and D). Data points represent the mean threshold of all participants, and error bars indicate the between-subjects 95% CI.

As shown in Figure 8A, resolution thresholds were resilient to simulated fixation instability. Again, contrast polarity reversal reduced thresholds, while temporal subsampling was detrimental to performance across the range of gain factors. A two-way ANOVA run on resolution thresholds revealed a significant main effect of temporal modulation condition, *F*(2, 10) = 116.9; *p* < 0.0001, a nonsignificant main effect of gain factor, *F*(5, 25) = 1.57; *p* = 0.21, and a significant interaction effect, *F*(10, 50) = 2.78; *p* = 0.0082. Figure 8B summarizes the changes in performance caused by contrast polarity reversal and temporal subsampling. Changes in performance were significant in all conditions but were somewhat smaller in magnitude at the highest gain factors. The results of associated *t* tests are shown in Table A5. Figures 8C and 8D show that a very similar pattern of performance was obtained when target position was restricted to an isoeccentric arc. However, a two-way ANOVA identified a significant main effect of gain factor in Experiment 3B, *F*(5, 25) = 4.62; *p* = 0.004, as well as a significant main effect of temporal modulation condition, *F*(2, 10) = 55.8; *p* < 0.0001) and an interaction effect, *F*(10, 50) = 5.09; *p* < 0.001. Post hoc analysis confirmed that this is driven by the reduction in threshold in temporal subsampling conditions at high gains: significant differences in resolution threshold between gain factors (within modulation condition) were only found for temporal subsampling. This analysis is shown in Table A7. Contrast polarity reversal, on the other hand, resulted in threshold improvements that were impervious to manipulations of eye movement gain.

## Discussion

The goal of this study was to investigate the potential to improve peripheral acuity by applying temporal modulation to a visual stimulus. We found that interleaving target presentation with blank periods (temporal subsampling) reduced the known detrimental effect of object motion at high speeds. However, a side effect of subsampling in this manner was a deterioration of acuity at low speeds. Contrary to these bidirectional effects, we found that periodically reversing the contrast polarity of the target improved resolution thresholds across a broad range of target speeds. This improvement was robust and consistent across conditions where the object translated along a smooth motion path, and when its position jittered over time in a manner consistent with the retinal motion introduced by fixational instability.

Why does temporal modulation affect performance at high speeds? Our data suggest that both forms of modulation essentially equate performance levels at higher speeds with those obtained with static or slowly moving targets. That is, they act to remove the typical pattern of speed-related loss observed with object motion. Loss of spatial acuity at high speed is generally attributed to motion smear, a blurring of the retinal image along the axis of motion (Burr 1980; Johnson & Casson, 1995; Watson & Ahumada, 2011). Accordingly, it is likely that the relative improvement in resolution thresholds found at high speeds in Experiments 1 and 2 reflects disruption of the motion signal and (at least partial) elimination of associated blur. Both temporal modulations investigated in the present study degrade the perception of motion, albeit in different ways. Temporal subsampling increases the spatial displacement between successive images (particularly at high speeds) making it harder for mechanisms that correlate information across space and time to detect motion (e.g., Braddick, 1974; Snowden & Braddick, 1989). Contrast polarity reversal disrupts the balance of motion energy in the stimulus, stimulating detectors tuned to the opposite direction of motion (Anstis & Mather, 1985; Anstis & Rogers, 1986). The ability to circumvent performance loss usually associated with dynamic targets could potentially be useful in real-world situations in which either the observer, or the object of interest, is in motion.

For static and slowly moving targets, the two forms of temporal modulation had dramatically different effects. Temporal subsampling progressively impaired resolution thresholds; the longer the gap between stimulus samples (the larger the ISI), the poorer the threshold. To test the hypothesis that deterioration in performance was driven by a reduction in stimulus visibility, we retested the different subsampled conditions after matching their time-averaged contrasts. Matching contrast in this manner resulted equated performance for subsampled static and slowly moving targets, while retaining the benefits observed for faster moving targets (Experiment 1B). Contrast polarity reversal, on the other hand, improved performance for static and slowly moving stimuli.

There are a number of candidate explanations for the improvement obtained with contrast polarity reversal at low speeds that warrant consideration. One possibility is that observers are able to benefit from the expansion of the spatiotemporal range of the stimulus—the temporal harmonics created by periodic modulation of the target (Van Santen & Sperling, 1985; Watson, 2013). This would require both that observers are sensitive to these higher temporal frequency signals and that the information contained is useful for the task at hand (i.e., discriminating target orientation). Analogous considerations are often made when choosing frame rates for capturing and displaying moving images. The nature of temporal sampling artefacts depends on both image speed and the temporal frequency at which it is sampled (Watson, 2013). Slow speeds induce low spatial and high temporal frequency artefacts (flicker), while faster speeds produce artefacts at high spatial and low temporal frequencies (spectral replicas). If either component falls within the human spatio-temporal contrast sensitivity window then they will be visible to the observer. The considerations for high-fidelity image display systems are to select parameters where the frame rate, capture and subsequent processing do not deliver additional information that is visible to the observer. In the case of contrast polarity reversal however, changes in the spectral content introduced by the temporal modulation may be beneficial to the observer. We have carried out some preliminary modeling to test the feasibility of this explanation. To date, we have confirmed that adding temporal modulation to a letter target stimulus alters its information content (indexed by the difference in spectral energy between orthogonal stimulus orientations) and its discriminability (the summed product of this difference spectrum and the psychophysical window of visibility; cf. Kelly, 1979). However, we are not yet in a position to provide a conclusive answer on the types of mechanisms required to fully account for all aspects of the data presented here. A useful avenue for experimentally testing this idea might be to alter the underlying sensitivity profile of the observer. For example, the spatial and temporal limits of contrast sensitivity are significantly lower for colored stimuli (Mullen, 1985). Therefore, by using isoluminant colored targets it should be possible to shrink the window of visibility while retaining the spectral profile arising from adding temporal modulation to the stimulus. If the performance benefits are driven by spectral changes that activate higher frequency mechanisms, the prediction would be that this information will now fall outside the window of visibility and the differences between contrast polarity reversal and unmodulated targets should disappear.

An alternative explanation is that by reversing contrast polarity, both on- and off-retinal mechanisms can contribute to target coding, leading to an improvement in resolution via some form of summation process (Schiller, Sandell, & Maunsell, 1986). Our data indicate that the benefits of this stimulus manipulation are lost at particularly high reversal rates (see 13.3 ms condition, Figure 6). This might be expected if insufficient time is available for on and off mechanisms to reach maximum output before each reversal. The temporal delay between stimulus onset and the associated neural response is known as the temporal response function. When stimuli appear within a receptive field for a shorter period than this, the receptor will fail to respond fully to the changes in stimulus intensity. When this response loss is associated with stimuli that are moving too quickly, it is referred to as motion blur and underlies the loss in resolution experienced at high speeds with unmodulated targets (Burr, 1980; Land, 1999). For static or slowly moving targets, the critical change becomes the switch in polarity rather than the rate of change in position over time. When the polarity switch is too rapid, the depth of the modulation will not be fully represented and contrast blur will be generated. Our data suggest that, like motion blur, this also causes a loss in resolution. The existence of different forms of blur is perhaps unsurprising. It is well known that for natural images at least, luminance and contrast information are statistically independent and separate gain mechanisms operate for each (Mante, Frazor, Bonin, Geisler, & Carandini, 2005). Relative performance for the different reversal rates in Figure 6 is broadly consistent with the temporal response functions of photoreceptor cells as estimated by Cao, Zele, and Pokorny (2007), Swanson, Ueno, Smith, and Pokorny (1987), and Zele, Cao, and Pokorny (2008).

It has recently been shown that adapting to fast flicker can enhance spatial acuity (Arnold, Williams, Phipps, & Goodale, 2016). In this study, subjects were exposed to dynamic noise masks for prolonged periods, after which their positional acuity and ability to read fine print were measured. Surprisingly, both acuity and reading showed significant improvement after adapting to fast flicker, but contrast sensitivity for low spatial frequencies was impaired. Is it possible that our contrast-polarity reversal condition engages the same mechanisms? The proposed explanation for the flicker adaptation effect is that information arising from coarse spatial channels is selectively suppressed via adaptation and that this suppression has the effect of removing blur from perception. Once low frequency information is removed, the remaining high frequency signals are perceptually sensitized, giving rise to superior acuity. This explanation is incompatible with the conditions investigated here, since the additional temporal modulations (polarity reversal and subsampling) are likely to selectively activate, rather than suppress, low spatial frequency mechanisms.

A final candidate mechanism is the Brucke-Bartley effect for luminance (see Bartley, 1938), or its recently described contrast analogue (Solomon & Tyler, 2018). This effect describes the relationship between the luminance of a stimulus and its perceptual representation (brightness). When subjects were asked to compare regions of a bipartite field, where one stimulus flickers and the other appears constant, nonlinear transduction produces a distortion product that makes the flickering field appear much brighter (Wu, Burns, Reeves, & Elsner, 1996). The effect is coupled to the intensity of the stimulus, such that flickering high intensity fields appear disproportionately bright (Wu et al., 1996). Solomon and Tyler (2018) have recently shown that for amplitude-modulated contrast, a similar effect is found. When contrast is modulated or flickered, apparent contrast is increased. The basis of this effect lies in an expansive nonlinearity in the transduction process. For contrast-modulated stimuli, the apparent contrast is enhanced across a broad range of temporal frequencies, with the effect dissipating around 30 Hz (Solomon & Tyler, 2018). Within this framework, it is possible that contrast polarity reversal increases the perceived brightness and contrast salience of targets when additional temporal modulations are introduced. This explanation would rely on a close link between resolution thresholds and apparent brightness and/or contrast of the target. Our primary results were obtained with full modulation (i.e., black to white and back), but we also found very similar thresholds when the depth of modulation was reduced (halved; see Figure 7). This makes it unlikely that flicker-induced changes in visibility explain the resolution improvements relative to unmodulated targets.

Experiment 3A and 3B both indicate that acuity is resistant to high levels of positional instability. The resistance to positional jitter is consistent with previous reports (Badcock & Wong, 1990; Falkenberg et al., 2007). However the exact mechanism of this phenomenon is unclear. Examined at the scale of individual frame transitions, stimulus speeds in Experiment 3 were comparable to the faster speed conditions in Experiments 1 and 2, where resolution was impaired. For instance, the jittered stimulus depicted in Figure 3 had a mean interframe target displacement of 0.37°, which corresponds to a speed of approximately 28°/s at the 75 Hz frame rate. However, this comparison does not take into consideration the frequent changes in direction in the jittered condition. One possibility is that resolution is only impaired when rapid image motion stimulates direction-selective mechanisms with relatively long temporal integration times. Further, both figures show a significant improvement in acuity for targets in the contrast polarity reversal conditions, even at high levels of positional jitter. Conversely, temporal subsampling consistently impaired resolution for the same conditions. The relative performance of the temporal subsampling and unmodulated conditions is perhaps not surprising, given the difference in overall energy (see Experiment 1B). The comparison, however, between unmodulated and polarity-reversing stimuli is of particular interest. The benefits of contrast polarity reversal, found for static and smoothly moving stimuli, also hold when significant positional jitter is introduced. This has important implications for patients with central vision loss resulting from macular disease, where fixation instability is typically magnified by a factor of 2× to 4× (Kumar & Chung, 2014). We find that thresholds remain largely constant for amplification of eye movements up to a factor of eight for contrast polarity reversing stimuli. Taking a different approach, where they compensated (or overcompensated) for abnormal fixational eye movements in patients with macular disease, Macedo et al. (2011) found that full compensation had little influence on acuity and thresholds only declined for very high levels of additional spatial jitter (overcompensation of eye movements by ×10). Given this, the acuity benefits of contrast polarity reversal should be realizable, even in patients with highly abnormal fixation patterns.

In order to ensure that eccentricity was not a confounding factor, in Experiments 1, 2, and 3B we held fixation constant and translated the target along an isoeccentric arc. While this was an important control for our study, it should be noted that under natural viewing conditions observers typically attempt to track moving targets with their eyes. For individuals with normal vision, smooth pursuit eye movements would undoubtedly improve performance, allowing the target to remain foveated. Understanding the effects of temporal modulation in patients with central vision loss during attempted tracking will be an important next step for translating the work presented here. Studies indicate that smooth pursuit is possible in patients with ARMD, but pursuit accuracy depends on several factors such as the position and degree of central loss and target trajectory with respect to the scotoma (Shanidze, Fusco, Potapchuk, Heinen, & Verghese, 2016; Shanidze, Heinen, & Verghese, 2017).

This study has demonstrated that temporal stimulus modulations are a robust and reliable technique for improving peripheral visual acuity. Contrast polarity reversal, in particular, has clear potential to enhance resolution across a broad range of visual conditions. The implications of our findings are that patients with severe central vision loss, who are forced to view objects peripherally, should benefit from the introduction of temporal modulations to visual stimuli that need to be resolved. Our data is a necessary first step toward the development of digital visual aids for use in patients with macular disease. Digital reading aids, such as tablet computers, have already been used for online stimulus manipulation (Walker, Bryan, Harvey, Riazi, & Anderson, 2016) and incorporating contrast polarity reversal may further optimize visual performance. Improving peripheral reading or other spatial tasks such as facial recognition, both of which are major functional problems for patients with macular disease, would be productive applications of contrast polarity reversal.

## Appendix 1: Full statistical tables

The following tables include details of the Student's *t* tests performed on threshold ratios. Negative mean difference implies a mean threshold ratio below 1; that is, resolution thresholds in that modulation condition were lower than the corresponding unmodulated threshold. Asterisks in the rightmost column indicate *p* values below the Bonferroni-corrected alpha score of 0.00833. Each test was two-tailed, reflecting the possibility of both positive and negative effects of modulation. Unless otherwise stated, group mean threshold ratios were tested against a threshold ratio of 1; the null hypothesis indicating no effect of additional temporal modulation on resolution thresholds (Tables A1–A6).

**Table A1:** Student's t test results from Experiment 1A.

Modulation condition	Speed (°/s)	Mean diff.	*SE* of diff.	*t*	*df*	*p*	Sig.
13.3 ms ISI	0	0.184	0.085	2.16	14	0.048	
	1.25	0.008	0.045	0.17	14	0.86	
	2.5	0.062	0.044	1.41	14	0.18	
	5	0.078	0.046	1.71	14	0.11	
	10	−0.064	0.040	1.58	14	0.14	
	20	−0.241	0.050	4.79	14	0.00028	*
26.7 ms ISI	0	0.349	0.124	2.81	14	0.014	
	1.25	0.123	0.046	2.69	14	0.018	
	2.5	0.130	0.070	1.86	14	0.085	
	5	0.062	0.091	0.69	14	0.50	
	10	−0.154	0.040	3.87	14	0.0017	*
	20	−0.277	0.041	6.78	14	8.89E-06	*
40.0 ms ISI	0	0.353	0.104	3.40	14	0.0043	*
	1.25	0.216	0.025	8.54	14	6.34E-07	*
	2.5	0.197	0.072	2.73	14	0.016	
	5	0.094	0.113	0.83	14	0.42	
	10	−0.083	0.053	1.56	14	0.14	
	20	−0.272	0.034	8.01	14	1.36E-06	*
66.7 ms ISI	0	0.641	0.206	3.11	14	0.0076	*
	1.25	0.416	0.056	7.42	14	3.27E-06	*
	2.5	0.346	0.116	3.00	14	0.0096	
	5	0.287	0.159	1.81	14	0.092	
	10	0.013	0.094	0.13	14	0.90	
	20	−0.187	0.042	4.46	14	0.00054	*

**Table A2:** Student's t test results from Experiment 1B.

Modulation condition	Speed (°/s)	Mean diff.	*SE* of diff.	*t*	*df*	*p*	Sig.
13.3 ms ISI	0	0.068	0.051	1.34	8	0.22	
	1.25	0.057	0.049	1.17	8	0.27	
	2.5	−0.005	0.013	0.40	8	0.70	
	5	−0.007	0.038	0.17	8	0.87	
	10	0.045	0.090	0.50	8	0.63	
	20	−0.193	0.041	4.68	8	0.0016	*
26.7 ms ISI	0	0.081	0.053	1.52	8	0.17	
	1.25	−0.011	0.041	0.28	8	0.79	
	2.5	−0.037	0.046	0.79	8	0.45	
	5	−0.075	0.026	2.86	8	0.021	
	10	−0.088	0.103	0.86	8	0.42	
	20	−0.328	0.022	14.87	8	4.10E-07	*
40.0 ms ISI	0	−0.020	0.086	0.24	8	0.82	
	1.25	−0.026	0.023	1.10	8	0.30	
	2.5	−0.046	0.036	1.18	8	0.27	
	5	−0.134	0.025	5.45	8	0.00061	*
	10	−0.138	0.098	1.41	8	0.20	
	20	−0.407	0.017	23.30	8	1.24E-07	*
66.7 ms ISI	0	−0.052	0.044	1.20	8	0.26	
	1.25	−0.071	0.043	1.64	8	0.14	
	2.5	−0.048	0.035	1.39	8	0.20	
	5	−0.140	0.040	3.50	8	0.0081	*
	10	−0.162	0.111	1.46	8	0.18	
	20	−0.430	0.033	12.89	8	1.24E-06	*

**Table A3:** Student's t test results from Experiment 2A.

Modulation condition	Speed (°/s)	Mean diff.	*SE* of diff.	*t*	*df*	*p*	Sig.
13.3 ms reversal	0	0.164	0.063	2.59	14	0.022	
	1.25	0.180	0.072	2.49	14	0.026	
	2.5	0.192	0.113	1.70	14	0.11	
	5	−0.002	0.056	0.04	14	0.97	
	10	−0.270	0.031	8.81	14	4.36E-07	*
	20	−0.353	0.036	9.74	14	1.28E-07	*
26.7 ms reversal	0	−0.072	0.050	1.43	14	0.17	
	1.25	−0.087	0.040	2.18	14	0.047	
	2.5	−0.072	0.052	1.39	14	0.19	
	5	−0.21	0.044	4.70	14	0.00034	*
	10	−0.345	0.049	7.01	14	6.17E-06	*
	20	−0.428	0.049	8.70	14	5.07E-07	*
40.0 ms reversal	0	−0.180	0.037	4.84	14	0.000260	*
	1.25	−0.176	0.036	4.95	14	0.000213	*
	2.5	−0.167	0.041	4.06	14	0.0012	*
	5	−0.273	0.026	10.35	14	6.06E-08	*
	10	−0.396	0.038	10.29	14	6.55E-08	*
	20	−0.448	0.047	9.62	14	1.51E-07	*
66.7 ms reversal	0	−0.220	0.030	7.40	14	3.35E-06	*
	1.25	−0.197	0.037	5.26	14	0.000121	*
	2.5	−0.200	0.051	3.97	14	0.0014	*
	5	−0.293	0.030	9.63	14	1.49E-07	*
	10	−0.369	0.041	9.06	14	3.15E-07	*
	20	−0.397	0.054	7.35	14	3.64E-06	*
106.7 ms reversal	0	−0.252	0.017	14.70	14	6.63E-10	*
	1.25	−0.154	0.021	7.46	14	3.078E-06	*
	2.5	−0.135	0.031	4.38	14	0.00062	*
	5	−0.258	0.036	7.23	14	4.35E-06	*
	10	−0.227	0.037	6.09	14	2.80E-05	*
	20	−0.329	0.040	8.24	14	9.76E-07	*
173.3 ms reversal	0	−0.216	0.012	17.74	14	5.41E-11	*
	1.25	−0.180	0.027	6.68	14	1.05E-05	*
	2.5	−0.167	0.056	2.99	14	0.0097	
	5	−0.202	0.038	5.33	14	0.00011	*
	10	−0.190	0.035	5.49	14	7.92E-05	*
	20	−0.309	0.042	7.30	14	3.94E-06	*

**Table A4:** Student's t test results from Experiment 2B.

Modulation condition	Speed (°/s)	Mean diff.	*SE* of diff.	*t*	*df*	*p*	Sig.
66.7 ms reversal, full contrast vs. unmodulated	0	−0.220	0.030	7.40	14	3.35E-06	*
	1.25	−0.197	0.037	5.26	14	0.00012	*
	2.5	−0.200	0.051	3.97	14	0.0014	*
	5	−0.293	0.030	9.63	14	1.49E-07	*
	10	−0.369	0.041	9.06	14	3.15E-07	*
	20	−0.397	0.054	7.35	14	3.64E-06	*
66.7 ms reversal, half contrast vs. unmodulated	0	−0.217	0.031	6.97	14	6.60E-06	*
	1.25	−0.176	0.051	3.45	14	0.0039	*
	2.5	−0.145	0.067	2.14	14	0.050	
	5	−0.237	0.057	4.18	14	0.00092	*
	10	−0.300	0.049	6.07	14	2.88E-05	*
	20	−0.345	0.056	6.15	14	2.51E-05	*
Full contrast vs. half contrast	0	−0.004	0.043	0.08	14	0.93	
	1.25	−0.021	0.063	0.34	14	0.74	
	2.5	−0.056	0.084	0.66	14	0.52	
	5	−0.056	0.064	0.87	14	0.40	
	10	−0.069	0.064	1.08	14	0.30	
	20	−0.052	0.078	0.67	14	0.51	

**Table A5:** Student's t test results from Experiment 3A.

Modulation condition	Speed (°/s)	Mean diff.	*SE* of diff.	*t*	*df*	*p*	Sig.
66.7 ms ISI	0	0.506	0.056	9.04	10	4.00E-06	*
	1.25	0.474	0.089	5.32	10	0.00034	*
	2.5	0.542	0.098	5.54	10	0.00025	*
	5	0.413	0.067	6.17	10	0.00011	*
	10	0.308	0.027	12.51	10	1.97E-07	*
	20	0.393	0.024	16.24	10	1.63E-08	*
66.7 ms reversal	0	−0.175	0.021	8.17	10	9.78E-06	*
	1.25	−0.172	0.051	3.41	10	0.0067	*
	2.5	−0.176	0.032	5.59	10	0.00023	*
	5	−0.121	0.037	3.28	10	0.0083	*
	10	−0.137	0.032	4.27	10	0.0016	*
	20	−0.125	0.017	7.50	10	2.06E-05	*

**Table A6:** Student's t test results from Experiment 3B.

Modulation condition	Speed (°/s)	Mean diff.	*SE* of diff.	*t*	*df*	*p*	Sig.
66.7 ms ISI	0	0.554	0.087	6.39	10	7.94E-05	*
	1.25	0.498	0.114	4.36	10	0.0014	*
	2.5	0.584	0.103	5.68	10	0.00020	*
	5	0.406	0.094	4.34	10	0.0015	*
	10	0.342	0.038	8.98	10	4.22E-06	*
	20	0.279	0.052	5.39	10	0.00031	*
66.7 ms reversal	0	−0.201	0.022	8.97	10	4.29E-06	*
	1.25	−0.186	0.043	4.30	10	0.0016	*
	2.5	−0.158	0.046	3.46	10	0.0062	*
	5	−0.155	0.043	3.59	10	0.0049	*
	10	−0.186	0.020	9.18	10	3.47E-06	*
	20	−0.137	0.032	4.33	10	0.0015	*

## Appendix 2: Post hoc analysis of Experiment 3B

Table A7 shows a list of pairs of conditions, the differences between which were significant. Tests were only done within modulation conditions. Analysis was performed using *t* tests.

**Table A7:** Post hoc analysis tests from Experiment 3B. ***p < 0.001; **p < 0.01; *p < 0.05.

Modulation condition	AGF 1	AGF 2	Mean difference in resolution thresholds (°)	Significance of difference
66.7 ms ISI	0	2	0.16	***
	0	4	0.14	***
	0	8	0.14	***
	0.5	2	0.11	**
	0.5	4	0.09	*
	0.5	8	0.08	*
	1	2	0.12	**
	1	4	0.10	**
	1	8	0.09	*
